# Investigating the Spatial Relationship between Social Vulnerability and Healthcare Facility Distribution in Nassau County, New York

**DOI:** 10.3390/ijerph20054353

**Published:** 2023-02-28

**Authors:** Alea Jones, Ijeoma Nnadi, Kelly Centeno, Giselle Molina, Rida Nasir, Gina G. Granger, Nicholas R. Mercado, Andrea A. Ault-Brutus, Martine Hackett, Ibraheem M. Karaye

**Affiliations:** 1 Pipeline Programs, Donald and Barbara Zucker School of Medicine at Hofstra/Northwell, Hempstead, NY 11549, USA; 2 Department of Population Health, Hofstra University, Hempstead, NY 11549, USA; 3 Department of Health Humanities and Bioethics, University of Rochester Medical Center, Rochester, NY 14642, USA

**Keywords:** healthcare access, social vulnerability, disparities, hotspot analysis, healthcare facility distribution, segregation, Nassau County, New York

## Abstract

Health is a fundamental human right, yet healthcare facilities are not distributed equitably across all communities. This study aims to investigate the distribution of healthcare facilities in Nassau County, New York, and examine whether the distribution is equitable across different social vulnerability levels. An optimized hotspot analysis was conducted on a dataset of 1695 healthcare facilities—dental, dialysis, ophthalmic, and urgent care—in Nassau County, and social vulnerability was measured using the FPIS codes. The study found that healthcare facilities were disproportionately distributed in the county, with a higher concentration in areas of low social vulnerability compared to areas of high social vulnerability. The majority of healthcare facilities were found to be clustered in two ZIP codes—11020 and 11030—that rank among the top ten wealthiest in the county. The results of this study suggest that socially vulnerable residents in Nassau County are at a disadvantage when it comes to attaining equitable access to healthcare facilities. The distribution pattern highlights the need for interventions to improve access to care for marginalized communities and to address the underlying determinants of healthcare facility segregation in the county.

## 1. Introduction

Access to quality and comprehensive healthcare is a fundamental human right designed to ensure the attainment and maintenance of the highest level of health among individuals and communities [1,2]. However, access to healthcare is not always equitable and is associated with disparities in the distribution of healthcare facilities across geographic regions [3,4,5,6]. This disparity can result in marginalized communities experiencing poor health outcomes, which contradicts the goal of the Healthy People 2030 initiative of eliminating health disparities and achieving health equity for all in the United States [2,5,7,8].

Nassau County, New York, has a history of racial and socioeconomic segregation that is associated with a disproportionate burden of health outcomes among racial and ethnic subgroups and across geographical locations [9]. For example, previous studies have shown that hospitalization rates for asthma and type 2 diabetes are two and a half times higher among non-Hispanic Black and Hispanic populations compared to non-Hispanic White populations in the county [10,11]. Similarly, the incidence rates of syphilis and chlamydia are five times as high in Nassau County among non-Hispanic Black and Hispanic populations, and the adolescent pregnancy rate is 47 per 1000 among non-Hispanic Blacks and Hispanics compared to 10 per 1000 among non-Hispanic Whites [10,11,12]. These diseases/health-related events can lead to a range of health complications, including ophthalmic, renal, oral, and emergent issues that may require specialized medical services available at healthcare facilities.

Despite the segregation and health disparities in Nassau County, information is lacking on the overall distribution of healthcare facilities in the county and whether these facilities are equitably distributed. In order to gain insight into how the physical distribution of the facilities can impact access to care for different communities in the county, an assessment of the county’s healthcare facility distribution is essential. This study aims to assess the spatial distribution of healthcare facilities—dental, ophthalmic, urgent care, and dialysis—in Nassau County and determine whether their clustering patterns vary by the social vulnerability of the geographical areas where they are located. Based on our understanding of the principles of equity and the social vulnerability index in Nassau County, we hypothesize that healthcare facilities would be distributed in a manner that aligns with the social vulnerability needs of the population. Specifically, we expect that spatial units with higher social vulnerability will have a higher concentration of health facility clusters than spatial locations with lower vulnerability. Our hypothesis is rooted in the expectation that an equitable distribution of healthcare facilities would require an allocation of resources based on need, with clusters of facilities in areas of high vulnerability and a dispersion of facilities in areas of low vulnerability. By testing this hypothesis, we aim to contribute to the existing literature on equity in healthcare and inform policy decisions directed at reducing healthcare disparities in Nassau County. The findings of this study can inform policymakers and healthcare providers about the patterns of healthcare access among different communities, which can help in the design of healthcare services and the equitable allocation of resources.

## 2. Materials and Methods

### 2.1. Data Sources

Healthcare facilities: A comprehensive list of healthcare facilities—dental, ophthalmic, urgent care, and dialysis—located in Nassau County was created using a Google search. The street address of each facility was obtained and geocoded for spatial analysis. The selection of dental, dialysis, ophthalmic, and urgent care facilities for this study was based on several factors. Firstly, these facilities are among the most commonly utilized healthcare services in the study area, and their inclusion, which totaled 1695 facilities, ensured that the results would be more representative of the county. Secondly, the use of optimized hotspot analysis requires a minimum of 30 incident data points to yield statistically meaningful results. By selecting these facilities, we were able to overcome limitations that could arise from using too few samples. Lastly, we specifically chose facilities that require in-person visits to spatially align their physical locations with the social vulnerability indices of the geographic locations in which they are located. This approach allowed us to accurately assess the physical proximity of these critical healthcare services to vulnerable populations in the study area, rather than those that can be accessed remotely through telemedicine or the postal mailing of drugs and biological samples from laboratories or pharmacies.

Social Vulnerability Index (SVI): The SVI data for Nassau County were abstracted from the Centers for Disease Control and Prevention’s Agency for Toxic Substances and Diseases Registry (ATSDR) [13]. Social vulnerability refers to the susceptibility of a community to external factors (such as natural and anthropogenic disasters) that may impact health [13]. SVI is a tool used to identify populations that may need support before, during, or after disasters [13]. The SVI data includes four themes: socioeconomic status, household characteristics, racial and ethnic minority status, housing type, and transportation. Each county is ranked on a continuous scale, with the highest vulnerability (90th percentile) scored as 1 and those below the 90th percentile as 0. Additional information regarding the Social Vulnerability Index is available elsewhere [13].

### 2.2. Spatial Statistical Analysis

Spatial joining was used to identify the healthcare facilities located within each ZIP code of the study area. The facility density per 100,000 was estimated as the total number of facilities in each ZIP code divided by the estimated total population in the ZIP code multiplied by 100,000. Choropleth maps were generated to illustrate the spatial distribution of healthcare facilities (per 100,000) by ZIP Code.

In order to assess the equitable distribution of healthcare facilities, we overlaid the SVI layer onto the facility layer and conducted an Optimized Hotspot Analysis (OHA) using fishnet polygons. OHA is a spatial statistical method used to assess the clustering or dispersion of features in a spatial area [14]. It examines the spatial data and determines the most suitable settings that will yield optimal results [14]. Specifically, in this study, the incident point data (healthcare facilities) were aggregated into weighted features, the distribution of which was assessed based on the most optimal spatial scale. The OHA tool employed the false discovery rate method to adjust for multiple testing and spatial dependence [14].

All spatial statistical analysis was conducted using ArcMap 10.7 (Esri ^®^, Redlands, CA, USA).

## 3. Results

**Figure 1:**
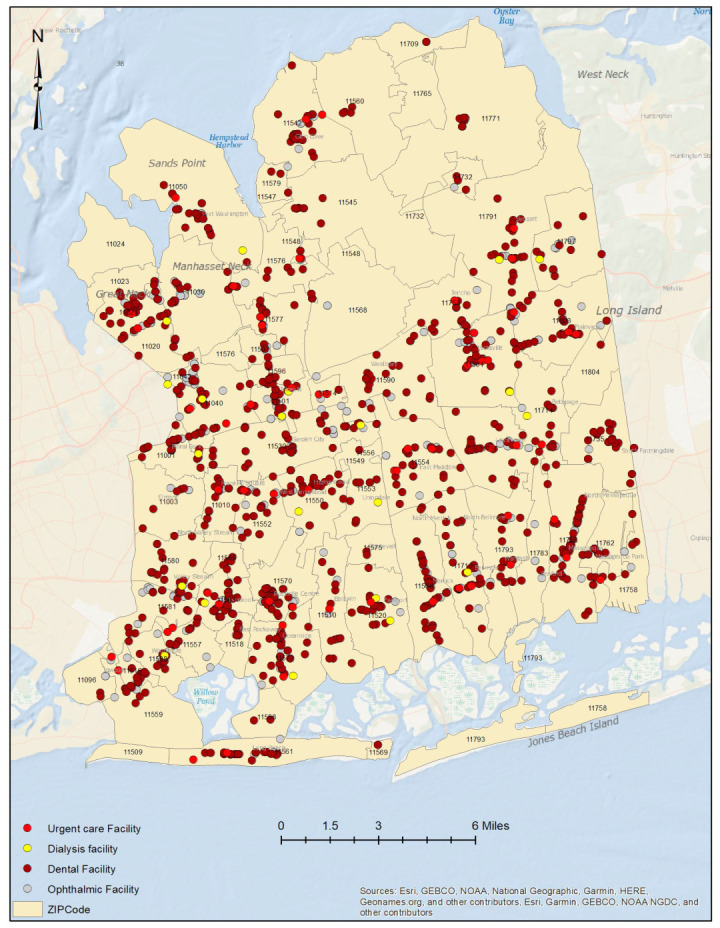
Spatial distribution of healthcare facilities in Nassau County, New York.

**Figure 2:**
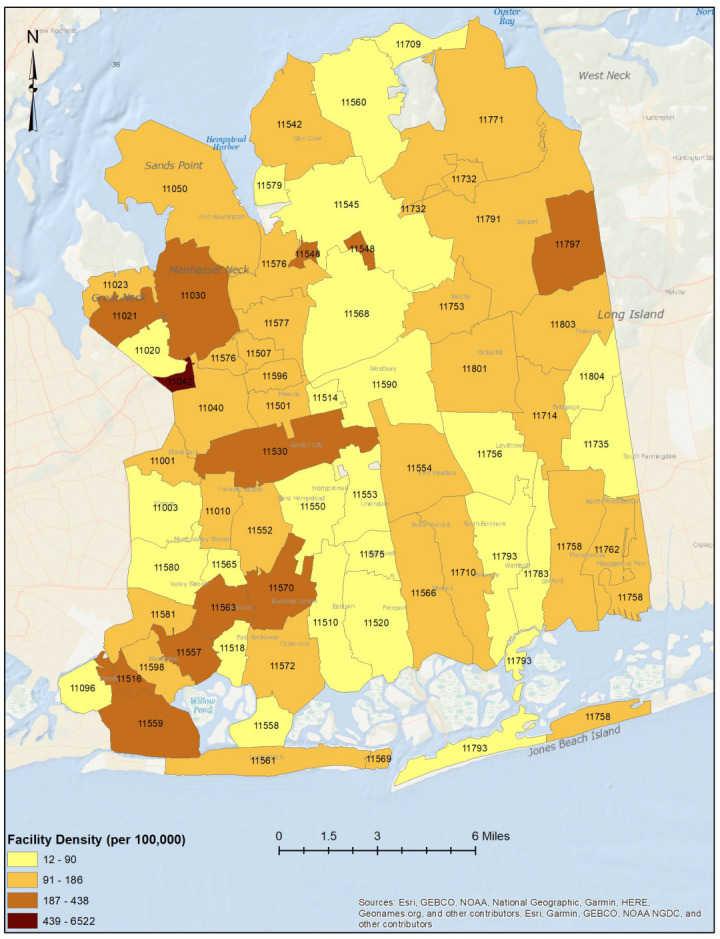
Choropleth map illustrating the spatial distribution of healthcare facilities (per 100,000) in Nassau County, New York.

A total of 1695 healthcare facilities were included in the study, comprising 1235 dental facilities, 23 dialysis facilities, 377 ophthalmic facilities, and 60 urgent care facilities. These facilities were distributed across the county, which has a total population of 1,390,907 (Figure 1, Figure 2 and Figure S1–S4).

### Spatial Analysis of Healthcare Facility Distribution in Relation to Social Vulnerability

An optimized hotspot analysis revealed that the healthcare facilities, when combined, were significantly clustered in geographical areas of low social vulnerability. Specifically, the ZIP codes 11,030 (which includes Manhasset, Plandome, Munsey Park, Flower Hill, North Hills, and Plandome Manor) and 11,020 (which includes Great Neck and Lake Success) had the highest concentration of facilities.

**Figure 3:**
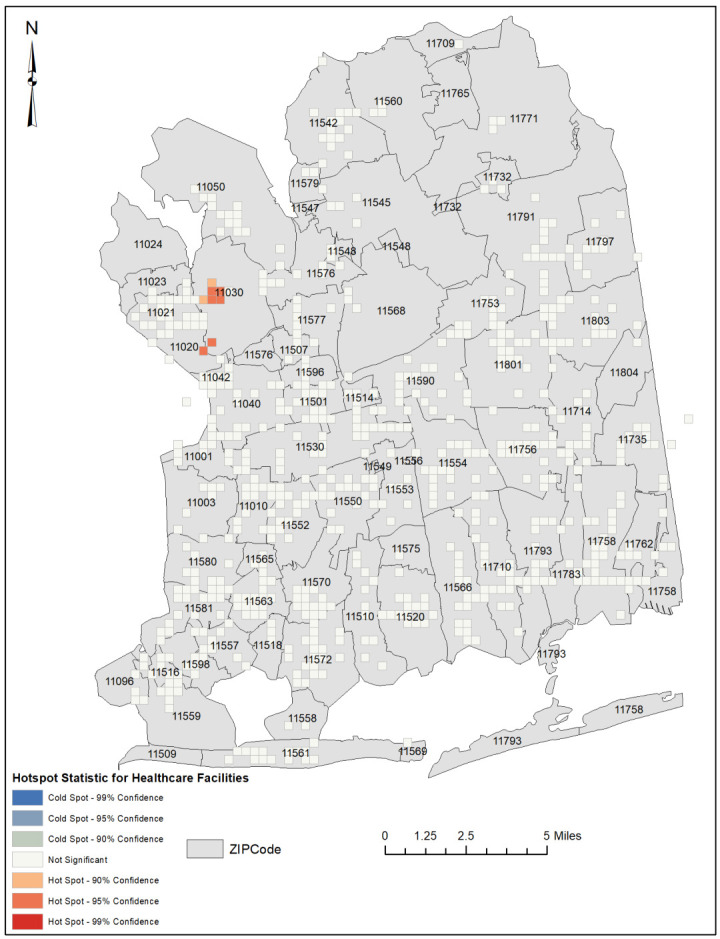
Hotspot map illustrating the spatial clustering of healthcare facilities by ZIP Code in Nassau County, New York.

**Figure 4:**
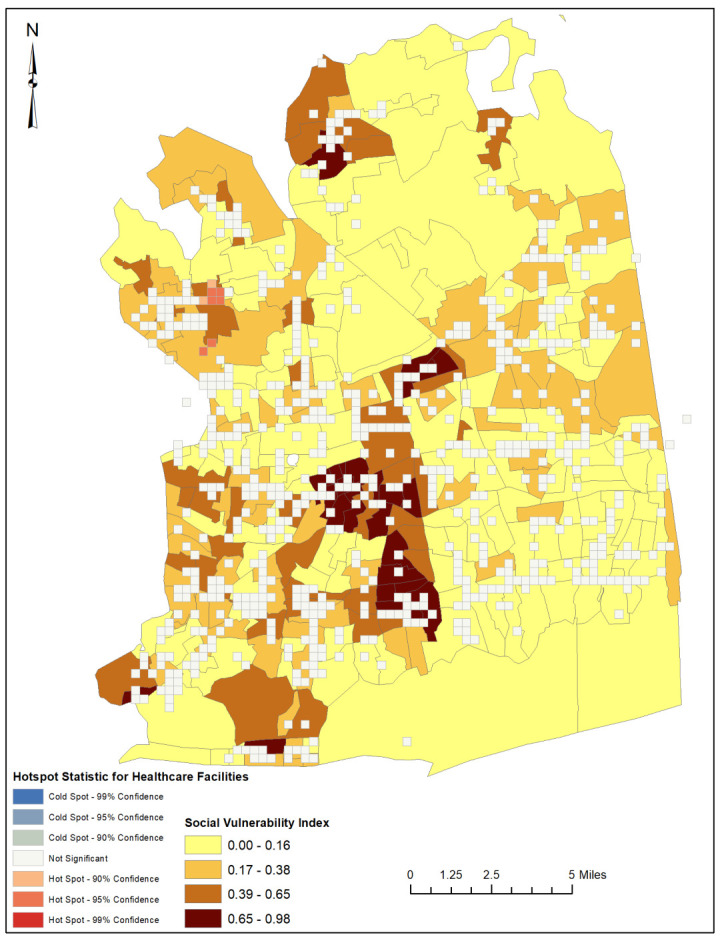
Hotspot map illustrating the spatial clustering of healthcare facilities in relation to social vulnerability in Nassau County, New York.

However, in the Northeastern part of the county, we observed an isolated pattern of sparsely distributed healthcare facilities that align with the low social vulnerability indices in this area, suggesting an equitable distribution of healthcare facilities. It should be noted that this finding is unique to this region and not reflective of the overall distribution of healthcare facilities in the county (Figure 3 and Figure 4).

However, when conducting hotspot analysis on each individual type of healthcare facility, the results revealed that the facilities were distributed uniformly across the study area rather than being concentrated in areas of high social vulnerability. This suggests that the distribution of healthcare facilities in Nassau County may not be equitable, as individual types of facilities are distributed uniformly rather than being targeted to areas of high social vulnerability. (Figures S5–S7)

It is worth noting that cluster analysis was not performed on dialysis facilities as they had fewer than 30 incident points, and the minimum requirement for incident points for OHA is 30, for reliable results [14].

## 4. Discussion

Healthcare facilities in Nassau County are disproportionately distributed, with a higher concentration in affluent areas compared to socially vulnerable areas. Specifically, the majority of healthcare facilities were found to be clustered in two ZIP Codes—11030 and 11020—that rank among the top ten wealthiest in the county [15]. This distribution pattern is concerning, as it suggests that socially vulnerable residents in the county are at a disadvantage when it comes to attaining equitable access to healthcare services. Furthermore, an examination of the individual types of healthcare facilities showed that the facilities were equally rather than equitably distributed in the study area, highlighting the need for further research to understand the underlying causes of this disparity and to develop strategies for addressing it. It is important to distinguish between equality and equity in our study. Equality refers to the even distribution of resources regardless of need, while equity requires resources to be allocated based on need. Our findings indicate that while the individual facility types were equally distributed throughout Nassau County, the distribution did not match the social vulnerability needs of the population. Therefore, the distribution of healthcare facilities in the county can be considered inequitable despite being equal in terms of the evenness of their distribution. An equitable distribution of healthcare facilities would require facility clusters in highly socially vulnerable locations and facility dispersion in areas of low social vulnerability.

Our findings align with a growing body of research on health inequities, which has shown that socioeconomic and racial/ethnic disparities exist in the distribution of healthcare facilities in some parts of the United States [5,16,17]. This leaves socially vulnerable communities with a disproportionately lower chance of accessing care. For example, a study by Eberth et al. [5] using data from the 2019 American Heart Association Annual Survey, found that areas with the largest concentration of minorities had disproportionately lower access to facilities that offered emergency, obstetric, trauma, surgical, and cardiac care, compared to areas with higher White representation.

The disparity in the distribution of healthcare facilities in Nassau County closely mirrors the racial and ethnic segregation in the county [9,18,19,20]. Nassau County is the tenth most segregated county in the United States and the fourth most segregated in New York [21]. This segregation is evident in the stark differences between neighboring townships such as Garden City and Hempstead, which are only separated by a few miles [22,23]. The median household income in Hempstead is nearly three times lower than that in Garden City, and the poverty rate is significantly higher [22,23].

An important first step to addressing the inequitable distribution of healthcare facilities in Nassau County is to identify the underlying determinants of residential segregation in the county. One of the key factors that has contributed to residential segregation in the county is the practice of “redlining” [20]. This practice, which was based on government-insured mortgages, involved ranking and mapping neighborhoods based on their loan worthiness. Neighborhoods where minorities lived were often determined to be risky investments and were marked red on the map to indicate that they would not qualify for lending programs [20]. Another factor that has contributed to residential segregation in Nassau County is the practice of racial steering [20,24]. This practice, which is conducted by realtors, involves directing potential buyers towards or away from a housing property based on their racial or ethnic status. Furthermore, “blockbusting,” which is a manipulative practice that involves introducing blacks to white neighborhoods in order to initiate a white exodus and a fall in house prices, also impacted residential segregation in the county [20].

Residential segregation is positively correlated with healthcare facility segregation [25,26,27,28,29,30]. Private healthcare facilities are more likely to be situated in areas of affluence due to social and economic factors such as fee-for-service-based reimbursement systems [26]. These systems make it more profitable for healthcare providers to operate in areas that are more affluent, as they reflect their desirable patient populations [26]. The result is healthcare segregation, with a concentration of healthcare facilities in areas that are more affluent and less in areas where minorities reside, disproportionately affecting socially vulnerable communities.

Our findings have important implications for policymakers and healthcare providers. The inequitable distribution of healthcare facilities in Nassau County highlights the need to improve access to care for marginalized communities. Interventions can include increasing funding for healthcare facilities in under-served areas, developing policies that increase the number of healthcare providers in low-income neighborhoods, incentivizing healthcare providers to establish facilities in under-served areas, and addressing other factors that impact healthcare access, such as transportation and language and cultural competence, to ensure that all residents have the information and resources they need to access healthcare services [31]. It is also important to note that improving healthcare facility distribution is not the only solution to healthcare access disparities. A comprehensive approach that addresses other factors—including health literacy, racism and discrimination, income, and health insurance, among others—may be needed to effectively improve healthcare access in Nassau County. The laudable pattern of equitable distribution of healthcare facilities in the northeastern segment of Nassau County provides a potential model that could be emulated to ensure more equitable distribution across the entire county. Future studies could focus on understanding the factors that led to this pattern, such as community engagement and targeted investment. Additionally, further research could explore the impact of equitable distribution of healthcare facilities on health outcomes in the area. The insights from these studies could inform policy decisions and guide resource allocation to address disparities in healthcare access in Nassau County.

This study has several limitations. The analysis of healthcare facility distribution was limited to Nassau County, and therefore, the results may not be applicable to other areas of Long Island, such as Suffolk, Kings, and Queens counties. In order to gain a more comprehensive understanding of healthcare access and equity, future studies should replicate this analysis in these areas. Additionally, this study only assessed the distribution of healthcare facilities and did not take into account the number of patients served by each facility or the quality of care provided. Further research should consider these factors to improve on this limitation. It should be noted that our study only considered a subset of healthcare facilities in Nassau County, specifically dental, dialysis, ophthalmic, and urgent care. Future research could expand upon this by including additional types of healthcare facilities, such as hospitals, pharmacies, and medical laboratories, amongst others. This would provide a more comprehensive understanding of the distribution of healthcare resources and the accessibility of care in the study area. Finally, this study only examined healthcare facility distribution as a measure of healthcare access. Other variables exist, including health insurance, transportation, health literacy, and income, that may also determine healthcare access and equity [32,33].

Despite these limitations, it is important to note that estimating healthcare facility distribution is an important first step in assessing healthcare access and equity, as validated by several studies within and outside the United [34,35,36,37]. It provides a baseline for understanding the spatial distribution of healthcare resources and identifying areas where access to healthcare may be limited.

## 5. Conclusions

Our study highlights that the distribution of healthcare facilities in Nassau County is inequitable and mirrors social segregation. To ensure that all residents have equitable access to healthcare services, it is essential to identify and address the underlying factors contributing to this inequity. Policy measures aimed at increasing the availability of healthcare services in underserved areas and improving the social conditions of vulnerable populations, including low-income individuals, those facing language barriers, and housing and transportation challenges, are necessary.

Additionally, to address this public health concern, we recommend that policymakers and stakeholders take a collaborative approach to improving the social and economic conditions in underserved areas. Specific measures could include the creation of incentives for healthcare providers to open or expand their practices in underserved areas, investment in transportation infrastructure to facilitate access to healthcare facilities, and the implementation of linguistically appropriate services for non-English speakers. Such efforts must be coupled with community engagement strategies that involve and empower the most affected populations.

Overall, addressing the underlying determinants of healthcare facility segregation is essential to ensuring equitable access to healthcare services for all residents of Nassau County.

## Data Availability

The data presented in this study are available on request from the corresponding author.

## Supplementary Materials

The following supporting information can be downloaded at: https://www.mdpi.com/article/10.3390/ijerph20054353/s1, Figure S1: Choropleth map illustrating the distribution of dental facilities (per 100,000 population) in Nassau County, New York; Figure S2: Choropleth map illustrating the distribution of dialysis facilities (per 100,000 population) in Nassau County, New York; Figure S3: Choropleth map illustrating the distribution of ophthalmic facilities (per 100,000 population) in Nassau County, New York; Figure S4: Choropleth map illustrating the distribution of urgent care facilities (per 100,000 population) in Nassau County, New York; Figure S5: Hotspot map illustrating the spatial distribution of dental facilities in relation to social vulnerability in Nassau County, New York; Figure S6: Hotspot map illustrating the spatial distribution of ophthalmic facilities in relation to social vulnerability in Nassau County, New York; Figure S7: Hotspot map illustrating the spatial distribution of urgent care facilities in relation to social vulnerability in Nassau County, New York.
